# Diagnostik und Therapie IgA Nephropathie – 2023

**DOI:** 10.1007/s00508-023-02257-6

**Published:** 2023-09-20

**Authors:** Judith Schimpf, Andreas Kronbichler, Martin Windpessl, Emanuel Zitt, Kathrin Eller, Marcus D. Säemann, Karl Lhotta, Michael Rudnicki

**Affiliations:** 1 Abteilung für Innere Medizin III (Nephrologie, Dialyse und Hypertensiologie), Akademisches Lehrkrankenhaus Feldkirch, Feldkirch, Österreich; 2 Department Innere Medizin IV (Nephrologie und Hypertensiologie), Medizinische Universität Innsbruck, Innsbruck, Österreich; 3 Abteilung für Innere Medizin IV, Klinikum Wels-Grieskirchen, Wels, Österreich; 4 Medizinische Fakultät, JKU Linz, Linz, Österreich; 5 Klinische Abteilung für Nephrologie, Abteilung für Innere Medizin III (Nephrologie, Dialyse und Hypertensiologie), Medizinische Universität Graz, Graz, Österreich; 6 6. Medizinische Abteilung mit Nephrologie & Dialyse, Klinik Ottakring, Wien, Österreich; 7 Medizinische Fakultät, SFU, Wien, Österreich; 8 Department Innere Medizin 4 (Nephrologie und Hypertensiologie), Medizinische Universität Innsbruck, Innsbruck, Österreich

**Keywords:** IgA Nephropathie, Proteinurie, Supportiven Therapie, SGLT2-Inhibitor, IgA Nephropathy, Proteinuria, Supportive therapy, SGLT2-inhibitor

## Abstract

Die Immunglobulin A Nephropathie (IgAN) ist die häufigste Glomerulonephritis weltweit und führt bei ungefähr einem Drittel der PatientInnen innerhalb von 10 bis 20 Jahren zur terminalen Nierenerkrankung. Die Pathogenese der IgAN ist nicht eindeutig geklärt. Vermutlich kommt es durch eine Dysregulation des mukosalen Immunsystems zu einer Untergalaktosylierung der IgA-Moleküle, Bildung von IgG Auto-Antikörpern und zur Zirkulation von IgG-IgA-Immunkomplexen, welche sich im Mesangium ablagern und schließlich zur Glomerulonephritis führen. Eine IgAN kann gelegentlich durch eine andere Erkrankung ausgelöst werden, deswegen sollten diese sekundären Ursachen einer IgAN identifiziert bzw. ausgeschlossen werden (chronisch entzündliche Darmerkrankungen, Infektionen, Tumore, rheumatische Erkrankungen). Charakteristische Befunde einer IgAN im variablen Ausmaß sind ein nephritisches Harnsediment (dysmorphe Erythrozyten, Akanthozyten, Erythrozytenzylinder), Proteinurie, eine Einschränkung der Nierenfunktion, eine arterielle Hypertonie, oder auch eine intermittierende schmerzlose Makrohämaturie, vor allem bei Infekten des oberen Respirationstraktes. Die Diagnose einer IgAN kann jedoch ausschließlich durch eine Nierenbiopsie gestellt werden. Dabei sollte eine histologische Klassifizierung (MEST‑C score) durchgeführt werden, um die Prognose abschätzen zu können. Die wichtigste therapeutische Maßnahme besteht in einer Optimierung der supportiven Therapie, das beinhaltet u. a. eine konsequente Kontrolle des Blutdrucks, eine Hemmung des RAS, und die Gabe eines SGLT2-Hemmers. Eine systemische immunsuppressive Therapie mit Kortikosteroiden wird kontroversiell diskutiert, sollte restriktiv gehandhabt und nur nach individueller Nutzen-Risiko-Abwägung unter bestimmten Bedingungen, die für eine progressive IgAN sprechen, verabreicht werden. Neue vielversprechende Therapeutika sind enterales Budesonid oder der duale Angiotensin-II-Rezeptor- und Endothelin-Rezeptor-Antagonist Sparsentan. Eine rapid-progressive IgAN sollte mit Kortikosteroiden und Cyclophosphamid ähnlich einer ANCA-assoziierten Vaskulitis behandelt werden.

## Abstract (en)

Immunoglobulin A nephropathy (IgAN) is the most common glomerulonephritis. It leads to end-stage kidney disease in about a third of the patients within 10 to 20 years. The pathogenesis of IgAN is incompletely understood. It is believed that a dysregulation of the mucosal immune system leads to undergalactosylation of IgA, followed by formation of IgG autoantibodies against undergalactosylated IgA, circulation of these IgG-IgA immune complexes, deposition of the immune complexes in the mesangium, ultimately resulting in glomerular inflammation. IgAN can occasionally be triggered by other diseases, these secondary causes of IgAN should be identified or ruled out (chronic inflammatory bowel disease, infections, tumors, rheumatic diseases). Characteristic findings of IgAN of variable extent are a nephritic urinary sediment (erythrocytes, acanthocytes, erythrocyte casts), proteinuria, impaired renal function, arterial hypertension, or intermittent painless macrohematuria, especially during infections of the upper respiratory tract. However, the diagnosis of IgAN can only be made by a kidney biopsy. A histological classification (MEST‑C score) should always be reported to be able to estimate the prognosis. The most important therapeutic measure is an optimization of the supportive therapy, which includes, among other things, a consistent control of the blood pressure, an inhibition of the RAS, and the administration of an SGLT2 inhibitor. A systemic immunosuppressive therapy with corticosteroids is discussed controversially, should be used restrictively and only administered after an individual benefit-risk assessment under certain conditions that speak for a progressive IgAN. New promising therapeutics are enteral Budesonide or the dual angiotensin-II-receptor- and endothelin-receptor-antagonist Sparsentan. Rapidly progressive IgAN should be treated with corticosteroids and cyclophosphamide like ANCA-associated vasculitis.

## Einleitung und Epidemiologie

Weltweit ist die IgA Nephropathie (IgAN) die häufigste primäre Glomerulonephritis mit einer Prävalenz von etwa 2,5/100.000 Einwohnern [1]. Auch wenn die Pathogenese noch nicht völlig geklärt ist, weisen große Schwankungen in der Prävalenz sowohl auf genetische als auch auf Umweltfaktoren in der Entstehung hin [2]. Männer sind etwa doppelt so häufig wie Frauen betroffen. Da die Erkrankung oft mit nur geringer klinischer Ausprägung und milden Urinbefunden verläuft, bleibt vermutlich ein Großteil der Patienten unentdeckt [3]. Dennoch wird etwa ein Drittel der Patienten innerhalb von 10 bis 20 Jahren terminal niereninsuffizient. Deshalb sollte bei allen Patienten nach bioptischer Diagnosesicherung unter anderem mithilfe der Bestimmung der Proteinurie, der errechneten glomerulären Filtrationsrate (eGFR) und der histopathologischen Klassifizierung (MEST‑C score) eine Prognoseabschätzung erfolgen [4]. Hauptbestandteil jeder Therapie ist eine optimale Supportivtherapie mit Behandlung einer arteriellen Hypertonie und Proteinurie [5]. Rezente Publikationen weisen zudem auf nephroprotektive Effekte der SGLT‑2 Hemmer hin [6]. Der Nutzen einer zusätzlichen immunsuppressiven Therapie wird kontrovers diskutiert und sollte aufgrund potenziell schwerer Nebenwirkungen nur nach Diskussion von Risiko und Benefit mit dem Patienten erwogen werden („*informed decision-making*“) [7, 8].

## Pathogenese

Genomweite Assoziationsstudien (GWAS) weisen auf eine genetische Komponente insbesondere in Genloci des MHC-Komplexes, von Wachstumsfaktoren, der intestinalen Mukosa-Abwehr sowie beim alternativen Komplementweg hin [2, 9]. Weiters spielt die Produktion von veränderten IgA1-Molekülen mit verminderter Galaktosylierung von Glykosyl-Seitenketten der Hinge-Region der schweren Kette (Galaktose-defizientes IgA1, Gd-IgA1) eine zentrale Rolle. Die Ursache dieser defekten Galaktosylierung ist nicht geklärt. Danach kommt es zur Bildung von IgG Auto-Antikörpern gegen Gd-IgA1, die zusammen mit Gd-IgA1 zirkulierende Immunkomplexe bilden, die sich im Mesangium der Nierenkörperchen ablagern [10, 11]. Als Folge werden der alternative Komplementweg sowie eine Proliferation von Mesangialzellen mit Freisetzung von Zytokinen (u. a. TNFα, IL‑6 und TGFβ) aktiviert, es resultieren vermehrte Bildung von mesangialer Matrix und letztlich glomeruläre und interstitielle Fibrose (siehe auch Histologie) [12]. Obwohl eine familiäre Häufung einer IgAN auftreten kann, konnte bisher kein einzelner zugrundeliegender Genlocus identifiziert werden. Pathogene Varianten der Kollagen Typ 4 Gene *COL4A3, COL4A4* und *COL4A5* bzw. Überlappungen mit dem Alport Syndrom, als auch dem Syndrom der dünnen Basalmembran wurden berichtet [13].

Eine sogenannte sekundäre IgAN kann auch in Zusammenhang mit verschiedenen anderen Erkrankungen auftreten (Tab. 1). Diese sollten bei allen Patienten mit IgAN ausgeschlossen werden [3]. Die IgA-Vaskulitis (ehemals Henoch-Schönlein-Purpura) stellt eine Variante mit systemischen Manifestationen wie Exanthem, Arthritis und gastrointestinalen Symptomen dar. Obwohl sie meist im Kindesalter auftritt, kann sie auch im Erwachsenenalter vorkommen [3].

**Table 1:** 

	Erkrankung
*Darm/Leber*	M. Crohn, Colitis ulcerosa, Zöliakie, Leberzirrhose
*Infektionen*	HBV, HCV, HIV, Tuberkulose, Staphylokokken (IgA-dominante infekt-assoziierte IgAN)
*Autoimmun*	Ankylosierende Spondylitis, rheumatoide Arthritis, Sjögren Syndrom, IgA Vaskulitis (Pupura Schönlein-Henoch)
*Malignome*	Bronchuskarzinom, Nierenzellkarzinom, Lymphom, IgA Myelom
*Lunge*	Sarkoidose, Bronchiolitis obliterans, zystische Fibrose
*Haut*	Dermatitis herpetiformis, Psoriasis

## Diagnostik und Pathologie

Da die IgAN über den Nachweis von glomerulären IgA Ablagerungen in der Immunhistochemie oder Immunfluoreszenz definiert ist, kann die Diagnose *ausschließlich* durch eine Nierenbiopsie gestellt werden. Neben den dominanten, überwiegend mesangialen mitunter auch entlang der Kapillarwände sichtbaren IgA Depots können häufig Komplement C3 und selten andere Komplementkomponenten (C4d, C1q), und/oder (in geringem Umfang) IgG nachweisbar sein. Beachte: Eine mesangiale IgA-Ablagerung kann auch in 3–16 % scheinbar gesunder Personen ohne klinische Zeichen für Nephritis vorkommen, sowie in nicht dominanter Form auch bei anderen Glomerulonephritis-Formen (v. a. Lupus Nephritis).

Die glomerulären Läsionsmuster der IgAN sind ebenso wie der klinische Verlauf sehr variabel und reichen von histologisch unveränderten Glomerula bis hin zur hochgradig aktiven nekrotisierenden GN mit Halbmonden (crescentic GN). Am häufigsten findet man jedoch das Bild einer mesangioproliferativen GN.

Vor rund 15 Jahren wurde in einem beispielhaften, mehrstufigen Analyseverfahren versucht, eine klinisch möglichst relevante histologische Klassifikation der IgAN zu etablieren, die als Oxford Klassifikation bekannt wurde. Dabei wurden in einer internationalen multizentrischen Studie systematisch jene histologischen Parameter identifiziert, die sowohl reproduzierbar quantifiziert werden konnten als auch prognostische Relevanz aufwiesen. Das in der initialen Publikation 2009 vorgestellte Scoring System wurde 2017 um die Kategorie extrakapilläre Proliferation/Halbmonde ergänzt, da die Relevanz dieser Läsion aufgrund der Designs der Originalstudie (Exklusion rasch progredienter Fälle) nicht analysiert werden konnte (Tab. 2; [14]). Die klinische Relevanz und die Reproduzierbarkeit des Scorings wurden in mehreren Studien nachgewiesen. Dennoch kann die Variabilität des klinischen Verlaufes der Erkrankung allein durch histologische und immunhistochemische Parameter weder umfassend erklärt noch verlässlich vorhergesagt werden [15].

**Table 2:** 

	MEST‑C score
*Mesangiale Hyperzellularität (M)*	
≤ 50 % der Glomerula	M0
> 50 % der Glomerula	M1
*Endokapilläre Hyperzellularität (E)*	
Nicht vorhanden	E0
Vorhanden	E1
*Segmentale Sklerose (S)*	
Nicht vorhanden	S0
Vorhanden	S1
*Tubuläre Atrophie/interstitielle Fibrose (T)*	
< 25 %	T0
26–50 %	T1
> 50 %	T2
*Halbmonde (crescents, C)*	
Keine	C0
≤ 25 % der Glomerula	C1
> 25 % der Glomerula	C2

Folgende Ergebnisse spezieller Laboruntersuchungen finden sich bei der IgAN:

- Im Harnsediment finden sich dysmorphe Erythrozyten und häufig Erythrozytenzylinder.
- Negative „Autoimmun-Serologie“ (ANA, PR3-/ MPO-AK, ggf. anti-GBM-AK, PLA2R-AK), kein Komplementverbrauch, Hepatitis B und C negativ (wenn einer dieser Parameter positiv ist, so ist eine andere Glomerulonephritis zu suszipieren!).
- Rolle von Serum IgA in der Diagnosestellung bzw. Monitoring ist unspezifisch. Die Bestimmung hat keine prognostische/diagnostische Relevanz (in 50 % der Fälle Serum IgA in der Norm).
- Noch unklare diagnostische Nützlichkeit und mangelnde Verfügbarkeit: Messung von Gd-IgA1 bzw. von Serumspiegeln von Anti-Gd-IgA1 IgG Antikörpern.

## Klinik

Die IgAN zeigt ein breites klinisches Spektrum [3]. In der Mehrzahl der Fälle findet sich im Rahmen einer Routineuntersuchung als Anlass zu weiterführender nephrologischer Abklärung:

- eine Mikrohämaturie
- eine Proteinurie

Möglich sind in 10–15 % aller Fälle:

- Rezidivierende Phasen einer selbstlimitierenden schmerzlosen Makrohämaturie, oft während oder kurz nach einem respiratorischen („synpharyngitisch“) oder gastrointestinalen Infekt.

Nur gelegentlich Anlass zur Diagnosestellung:

- eingeschränkte Nierenfunktion (eGFR)
- arterielle Hypertonie

Seltene Verlaufsformen einer IgAN:

- **Nephrotisches Syndrom** (Proteinurie > 3,5 g/Tag): Diese zeigt im histologischen Bild einen zusätzlichen Podozytenschaden wie bei einer Minimal Change Nephropathie [16].
- **Rapid-progressive Glomerulonephritis** (RPGN): Die RPGN beinhaltet einen glomerulären Filtrationsverlust von ≥ 50 % innerhalb von drei Monaten und hat eine schlechte Prognose. Histopathologisch findet sich häufig eine Halbmondbildung in > 50 % der Glomeruli. Sie kann sowohl zum Diagnosezeitpunkt aber auch jederzeit im Laufe der Erkrankung auftreten.
- **Akute Nierenschädigung** durch Tubulusobstruktion und -toxizität kann im Rahmen von Makrohämaturie-Episoden auftreten, bessert sich zumeist binnen weniger Wochen.

*Alle Patienten sollten regelmäßig auf Nierenfunktion (eGFR), Proteinurie und Blutdruck kontrolliert werden, da auch bei initial milden Verlaufsformen ein fortschreitender Nierenfunktionsverlust auftreten kann *[17, 18]*!*

## Prognose

Die Prognose der IgAN ist sehr variabel. Wichtige klinische Prognoseparameter sind:

- Nierenfunktion (eGFR) zum Zeitpunkt der Diagnose
- Proteinurie
- Arterielle Hypertonie

sowie

- Isolierte Hämaturie und keine Proteinurie: niedriges Risiko für Progression
- Signifikante persistierende Proteinurie **>** **1** **g/24** **h**: 25–30 % dieser Patienten sind dialysepflichtig innerhalb von 20–25 Jahren
- Spontane Remission möglich bei isolierter Hämaturie (ohne signifikante Proteinurie und mit normaler Nierenfunktion, v. a. bei Kindern (5–30 %))
- Reduktion der Progressionsrate durch Erreichen einer kompletten (Proteinurie < 0,3 g/d) oder partiellen Remission (Proteinurie < 1 g/d)

Laut Literatur erreichen nach zehn Jahren 15–25 % und nach 20 Jahren 20–30 % aller IgAN-Patienten eine terminale Niereninsuffizienz [3]. Auch im Langzeitverlauf der STOP-IgAN Studie mit optimaler supportiver Therapie über mehr als sieben Jahre betrug der Anteil der Patienten, die den kombinierten Endpunkt aus GFR-Verlust > 40 %, Dialysepflicht oder Tod erreichten, 50 %, und 25 % der Patienten wurden dialysepflichtig [19]. Dabei betrug der mittlere eGFR Verlust −2,5 ml/min/1,73 m^2^ pro Jahr. Selbst beim sogenannten benignen Phänotyp, d. h. einer eGFR > 60 ml/min/1,73 m^2^, einer milden Proteinurie von ca. 0,3 g/24 h und einer Mikrohämaturie zum Zeitpunkt der Diagnosestellung, zeigen 18,6 % der Patient:innen bei einer Nachbeobachtung von mehr als 20 Jahren einen eGFR Abfall von > 50 %, und 2,8 % werden dialysepflichtig [18]. Insofern sollte auch diesen Patient:innen eine regelmäßige nephrologische Kontrolle angeboten werden.

Bei jedem Patienten mit IgAN wird empfohlen, zum Zeitpunkt der Biopsie die Prognose hinsichtlich eGFR-Verlust > 50 % oder terminaler Niereninsuffizienz über bis zu 80 Monate mit Hilfe des International Risk-Prediction Tool in IgA Nephropathy (siehe https://qxmd.com/calculate/) anhand klinischer Daten und des MEST‑C Scores abzuschätzen [4].

## Therapie

Da derzeit eine kausale Therapie der IgAN unbekannt ist, stützt sich die Behandlung vorwiegend auf allgemeine Maßnahmen der Progressionshemmung [5]. Diese sind in Tab. 3 zusammengefasst.

**Table 3:** 

ACE-Hemmer oder Sartan in maximal verträglicher Dosis
Blutdrucksenkung: systolischer Zielblutdruck < 130 mm Hg bzw. < 120 mm Hg wenn tolerierbar
SGLT2-Hemmer
Kochsalzeinschränkung auf < 5 g/d (Natrium < 2 g/d)
Statintherapie bei Alter > 50 Jahre oder Cholesterin > 200 g/dl
Optimierung des Glukose-Metabolismus
Übergewicht reduzieren (Ziel BMI ≤ 25 kg/m^2^)
Nikotinkarenz
Nephrotoxische Substanzen (z. B. NSAR) vermeiden

**Figure 1:**
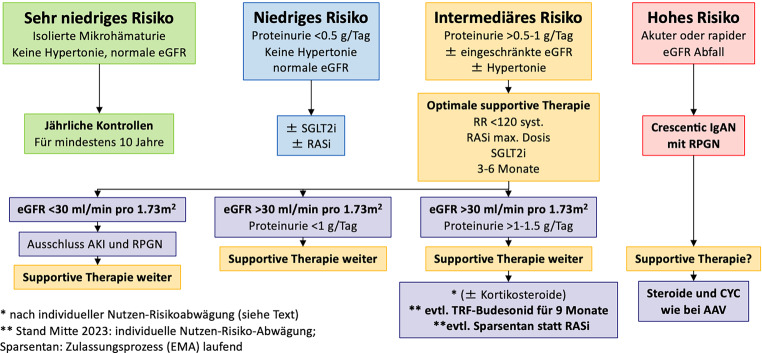

Therapeutisches Ziel ist es, durch diese Maßnahmen die Proteinurie zumindest unter 1 g/Tag, besser unter 0,5 g/Tag zu senken (siehe Abb. 1).

- *Sehr niedriges Risiko der Progression:* PatientInnen mit isolierter Hämaturie, ohne Hypertonie und normaler eGFR benötigen für gewöhnlich keine spezifische Therapie, jedoch zumindest jährliches Monitoring der Proteinurie, der eGFR und des Blutdrucks.
- *Niedriges Risiko der Progression:* Bei einer Proteinurie < 0,5 g/Tag sollte die Entscheidung für eine RAS- bzw. eine SGLT2-Blockade individuell getroffen werden (je nach Blutdruck und Komorbiditäten).
- *Intermediäres Risiko der Progression:* Bei PatientInnen mit persistierender Proteinurie von über 0,5 g/Tag sollte eine Therapie mit einem ACE-Hemmer oder einem Sartan begonnen werden mit dem Ziel die Proteinurie < 0,5 g/Tag zu reduzieren und den Blutdruck auf zumindest < 120 mm Hg systolisch zu senken. Die Dosis der RAS-Blockade sollte bis zur maximal tolerierbaren bzw. zugelassenen Dosierung titriert werden. In der STOP-IgAN Studie konnte durch supportive Therapie der jährliche eGFR Verlust von −5 bis −6 ml/min/1,73 m^2^ pro Jahr auf −1,6 ml/min/1,73 m^2^ pro Jahr reduziert werden [8]. In einer Subanalyse der IgAN Patient:innen in der DAPA-CKD Studie konnte gezeigt werden, dass durch den SGLT2-Hemmer Dapagliflozin 10 mg täglich das Risiko für rasche Progression, Dialysepflichtigkeit oder Tod innerhalb von 2 Jahren um 70 % reduziert wird [6]. Der nephroprotektive Effekt von SGLT2-Hemmern bei Patienten mit und ohne Diabetes konnte in der EMPA-KIDNEY Kohorte (ca. 25 % Patient:innen mit glomerulären Erkrankungen) bestätigt werden [20]. Daher scheint die zusätzliche Gabe eines SGLT2-Hemmers für viele IgAN Patient:innen bis zu einer eGFR von 20 ml/min/1,73 m^2^ sinnvoll. Eine Reevaluation von Nierenfunktion und Proteinurie sollte nach 3–6 Monaten erfolgen. Nur im Fall einer eGFR > 30 ml/min/1,73 m^2^ und einer Proteinurie > 1 bis 1,5 g/Tag sollten weitere therapeutische Optionen mit dem Patienten individuell diskutiert werden (siehe Absatz Immunsuppression).

### Immunsuppression

Der Langzeitnutzen einer Steroidtherapie zusätzlich zu den oben angeführten Allgemeinmaßnahmen ist zweifelhaft [5, 8]. Gemäß den aktuellen KDIGO Richtlinien zum Management glomerulärer Erkrankungen sollte eine Steroidtherapie bei IgAN prinzipiell nur bei persistierender Proteinurie > 1 g/d trotz 6‑monatiger optimaler konservativer Therapie und weitgehend erhaltener Nierenfunktion (eGFR zumindest ≥ 30 ml/min pro 1,73 m^2^) erwogen werden [5]. Es wird nicht empfohlen, eine Therapieentscheidung für eine immunsuppressive Therapie anhand des MEST‑C scores oder des Progressionrisikos (berechnet mittels des International IgAN Prediction Tools) zu treffen. Allerdings könnten bestimmte Patienten mit „aktiven Läsionen“ (M1, E1, C2) von einer Steroidtherapie profitieren [21].

Bei folgenden Befunden sollten Steroide möglichst vermieden werden:

- eGFR < 30 ml/min pro 1,73 m^2^
- Diabetes
- Adipositas (BMI > 30 kg/m^2^)
- Latente Infektionen (Hepatitis B und C, Tuberkulose)
- Sekundäre Erkrankungen (z. B. Leberzirrhose)
- Peptische Ulzera
- Unkontrollierte psychiatrische Erkrankung
- Schwere Osteoporose

Zur Steroiddosis bei IgAN wurden unterschiedliche Therapieregime publiziert (Methylprednisolon 0,6–0,8 mg/kg pro Tag und Prednison 0,8–1 mg/kg pro Tag) mit Variationen in der Dauer und im Tapering (zusammengefasst in den KDIGO Richtlinien zum Management glomerulärer Erkrankungen [5]). Aufgrund des erheblichen Nebenwirkungsprofils dieser Dosierungen auf der einen Seite und rezenter positiver Daten zum TESTING low-dose Methylprednisolon Schema auf der anderen Seite, empfehlen wir in den oben genannten Fällen folgendes Niedrigdosis-Regime:

Methylprednisolon 0,4 mg/kg pro Tag (maximal 32 mg) für 2 Monate, danach Reduktion um 4 mg pro Tag pro Monat über 6–9 Monate [22].

Auf eine konsequente Ulcus- und Osteoporoseprophylaxe ist zu achten.

Der fragliche Nutzen einer Steroidtherapie als auch typische Nebenwirkungen wie schwere Infekte, Diabetes mellitus, Osteoporose und Gewichtszunahme, müssen mit den PatientInnen diskutiert werden („*informed decision-making*“) [7, 8].

Die Behandlung der rapid progressiven IgAN erfolgt in Analogie zur ANCA-assoziierten Vaskulitis mit Cyclophosphamid und Steroiden. Entsprechende Therapieschemata sind im Beitrag über ANCA-assoziierte Vaskulitiden angeführt. Zu beachten ist, dass das Vorhandensein von Halbmonden (= Crescents) bei unauffälligem Serum Kreatinin bzw. Kreatinin-Verlauf nicht automatisch eine RPGN bedeutet! Diese PatientInnen sollten jedoch engmaschig kontrolliert und eventuell auch rebiopsiert werden. Halbmonde in ≤25 % der Glomeruli (MEST‑C score C1) während Episoden von Makrohämaturie und akuter Verschlechterung der Nierenfunktion bei IgAN sind möglich und lösen sich bei den meisten Patienten ohne bzw. mit wenig Narbenbildung wieder auf, insbesondere wenn die „Crescents“ vorwiegend zellulär sind (ohne signifikante Fibroblasten- oder Kollagenkomponente).

Die nephrotische Verlaufsform mit Nachweis von podozytären Fußfortsatzverschmelzungen wird wie eine Minimal Change Nephropathie therapiert (Therapieempfehlung im entsprechenden Beitrag).

### Mögliche zukünftige Therapieansätze

Targeted-release formulation (TRF) Budesonid wird im distalen Ileum, dem Ort der Gd-IgA1-Produktion, freigesetzt. In der NEFIGAN Studie konnten durch 8 bis 16 mg TRF Budesonid täglich die Proteinurie (−25 %) gesenkt und die eGFR stabilisiert werden [23]. Eine Interimsanalyse der NefIgArd Phase 3 Studie (Teil A) zeigte einen positiven Effekt auf die eGFR (3,87 ml/min/1,73 m^2^ Unterschied zu Placebo) sowie eine Reduktion der Proteinurie um 27 % [24]. Ergebnisse des Teils B der Studie wird uns über Langzeiteffekte informieren. Basierend auf diesen Daten wurde Nefecon durch die FDA (12/21) sowie die EMA (07/22) zugelassen.

Als off-label use ist Hydroxychloroquin überlegenswert, insbesondere bei asiatischen IgAN Patienten mit hohem Risiko für Progression [25].

- Bei eGFR > 30 ml/min, Proteinurie > 750 mg/die
- Dosierung 100–400 mg/Tag je nach eGFR über mind. 6 Monate
- Beachtung der Kontraindikationen und notwendigen Kontrollen (Auge, Leber)

Ein anderer neuer therapeutischer Ansatz bei der IgAN ist die Endothelin-Rezeptor- bzw. die kombinierte Endothelin-Rezeptor- und Angiotensin-II-Rezeptor-Blockade. Phase 3 Studien zur Wirkung von Atrasentan und Sparsentan zeigen vielversprechende Zwischenergebnisse bzgl. der Proteinuriereduktion.

Einen weiteren Angriffspunkt in klinischer Erprobung stellen Hemmer des Komplementsystems dar (Iptacopan, Cemdisiran). Auch bei diesen Substanzen zeigen nicht-publizierte Zwischenergebnisse der Zulassungsstudien günstige Effekte auf die Proteinurie.

## Transplantation

Etwa ein Viertel der nierentransplantierten PatientInnen mit IgAN entwickelt innerhalb von 15 Jahren eine Rekurrenz im Nierentransplantat. Dies geht mit einem erhöhten Risiko für ein Transplantatverlust einher [26]. Klinische Zeichen dafür sind eine neu auftretende Mikrohämaturie und Proteinurie. Auch hier ist vermutlich, neben der Basis-Immunsuppression, nur die Intensivierung der supportiven Therapie angebracht.

## Schwangerschaft

Bei initial guter Nierenfunktion ist keine renale Progression bei IgAN-Patientinnen zu erwarten, jedoch ist das Risiko für Präeklampsie/Eklampsie sowie Frühgeburten erhöht [27].

Wir empfehlen daher:

- Optimale Blutdruckkontrolle unter Verwendung der in der Schwangerschaft etablierten Medikamente
- Regelmäßige (alle 6 Wochen) Bestimmung von Nierenfunktion (eGFR), Harnbefund inkl. Sediment sowie Bestimmung der Proteinurie
- Engmaschige gynäkologische Kontrollen

## Verlaufskontrollen

- Alle Patienten mit IgAN bedürfen einer nephrologischen Verlaufskontrolle (siehe Abb. 1).
- Patienten mit Proteinurie < 1 g/24 h, normaler eGFR ± Mikro/Makrohämaturie: alle 6 Monate (eGFR, Harnsediment, Protein/Kreatinin-Ratio, Blutdruck).
- Bei Einleitung einer supportiven Therapie zumindest Kontrollen in zweimonatigen Abständen bis zum Erreichen des Therapiezieles.
- Bei relevanter Änderung der Protein/Kreatinin Ratio, Proteinurie im 24-Stunden Harn bestimmen lassen.
- Evaluierung Nierenbiopsie bzw. Re-Biopsie bei nicht durch alternative Ursachen erklärbarer Verschlechterung der eGFR in Kombination mit nephritischem Sediment.
- Verlaufskontrollen bei Patienten, die unter systemischer Glukokortikoidtherapie stehen, zumindest alle 4 bis 6 Wochen (Infekt-Screening, metabolische Nebenwirkungen).

## References

[1] McGrogan A, Franssen CF, de Vries CS (2011). The incidence of primary glomerulonephritis worldwide: a systematic review of the literature. Nephrol Dial Transplant.

[2] Gharavi AG, Kiryluk K, Choi M, Li Y, Hou P, Xie J (2011). Genome-wide association study identifies susceptibility loci for IgA nephropathy. Nat Genet.

[3] Pattrapornpisut P, Avila-Casado C, Reich HN (2021). IgA nephropathy: core curriculum 2021. Am J Kidney Dis.

[4] Barbour SJ, Coppo R, Zhang H, Liu ZH, Suzuki Y, Matsuzaki K (2019). Evaluating a new international risk-prediction tool in IgA nephropathy. JAMA Intern Med.

[5] Kidney Disease: Improving Global Outcomes Glomerular Diseases Work Group (2021). KDIGO 2021 clinical practice guideline for the management of glomerular diseases. Kidney Int.

[6] Wheeler DC, Toto RD, Stefansson BV, Jongs N, Chertow GM, Greene T (2021). A pre-specified analysis of the DAPA-CKD trial demonstrates the effects of dapagliflozin on major adverse kidney events in patients with IgA nephropathy. Kidney Int.

[7] Tesar V, Troyanov S, Bellur S, Verhave JC, Cook HT, Feehally J (2015). Corticosteroids in IgA nephropathy: a retrospective analysis from the VALIGA study. J Am Soc Nephrol.

[8] Rauen T, Eitner F, Fitzner C, Sommerer C, Zeier M, Otte B (2015). Intensive supportive care plus immunosuppression in IgA nephropathy. N Engl J Med.

[9] Feehally J, Farrall M, Boland A, Gale DP, Gut I, Heath S (2010). HLA has strongest association with IgA nephropathy in genome-wide analysis. J Am Soc Nephrol.

[10] Kiryluk K, Novak J (2014). The genetics and immunobiology of IgA nephropathy. J Clin Invest.

[11] Tomana M, Novak J, Julian BA, Matousovic K, Konecny K, Mestecky J (1999). Circulating immune complexes in IgA nephropathy consist of IgA1 with galactose-deficient hinge region and antiglycan antibodies. J Clin Invest.

[12] Suzuki H, Kiryluk K, Novak J, Moldoveanu Z, Herr AB, Renfrow MB (2011). The pathophysiology of IgA nephropathy. J Am Soc Nephrol.

[13] Savige J, Harraka P (2021). Pathogenic variants in the genes affected in alport syndrome (COL4A3-COL4A5) and their association with other kidney conditions: a review. Am J Kidney Dis.

[14] Trimarchi H, Barratt J, Cattran DC, Cook HT, Coppo R, Haas M (2017). Oxford classification of IgA nephropathy 2016: an update from the IgA nephropathy classification working group. Kidney Int.

[15] Rodrigues JC, Haas M, Reich HN (2017). IgA nephropathy. Clin J Am Soc Nephrol.

[16] Herlitz LC, Bomback AS, Stokes MB, Radhakrishnan J, D’Agati VD, Markowitz GS (2014). IgA nephropathy with minimal change disease. Clin J Am Soc Nephrol.

[17] Szeto CC, Lai FM, To KF, Wong TY, Chow KM, Choi PC (2001). The natural history of immunoglobulin a nephropathy among patients with hematuria and minimal proteinuria. Am J Med.

[18] Knoop T, Vikse BE, Mwakimonga A, Leh S, Bjorneklett R (2017). Long-term outcome in 145 patients with assumed benign immunoglobulin A nephropathy. Nephrol Dial Transplant.

[19] Rauen T, Wied S, Fitzner C, Eitner F, Sommerer C, Zeier M (2020). After ten years of follow-up, no difference between supportive care plus immunosuppression and supportive care alone in IgA nephropathy. Kidney Int.

[20] Group E‑KC, Herrington WG, Staplin N, Wanner C, Green JB, Hauske SJ, et al. Empagliflozin in patients with chronic kidney disease. N Engl J Med. 2022;388(2):117–27. 10.1056/NEJMoa2204233 PMC7614055 36331190

[21] Troyanov S, Hladunewich MA, Reich HN (2022). How should pathology findings influence treatment in IgA nephropathy?. Kidney Int Rep.

[22] Lv J, Wong MG, Hladunewich MA, Jha V, Hooi LS, Monaghan H (2022). Effect of Oral Methylprednisolone on Decline in Kidney Function or Kidney Failure in Patients With IgA Nephropathy: The TESTING Randomized Clinical Trial. JAMA.

[23] Fellstrom BC, Barratt J, Cook H, Coppo R, Feehally J, de Fijter JW (2017). Targeted-release budesonide versus placebo in patients with IgA nephropathy (NEFIGAN): a double-blind, randomised, placebo-controlled phase 2b trial. Lancet.

[24] Barratt J, Lafayette R, Kristensen J, Stone A, Cattran D, Floege J (2022). Results from part A of the multi-center, double-blind, randomized, placebo-controlled NefIgArd trial evaluated targeted-release formulation of budesonide for the treatment of primary immunoglobulin A nephropathy. Kidney Int..

[25] Liu LJ, Yang YZ, Shi SF, Bao YF, Yang C, Zhu SN (2019). Effects of hydroxychloroquine on proteinuria in IgA nephropathy: a randomized controlled trial. Am J Kidney Dis.

[26] Uffing A, Perez-Saez MJ, Jouve T, Bugnazet M, Malvezzi P, Muhsin SA (2021). Recurrence of IgA nephropathy after kidney transplantation in adults. Clin J Am Soc Nephrol.

[27] Liu Y, Ma X, Zheng J, Liu X, Yan T (2016). A systematic review and meta-analysis of kidney and pregnancy outcomes in IgA nephropathy. Am J Nephrol.

